# Outside-in technique versus inside-out semitendinosus graft harvest technique in ACLR: a randomised control trial

**DOI:** 10.1186/s43019-022-00144-4

**Published:** 2022-03-28

**Authors:** Silvampatti Ramasamy Sundararajan, Rajagopalakrishnan Ramakanth, Amit Kumar Jha, Shanmuganathan Rajasekaran

**Affiliations:** 1 Department of Arthroscopy and Sports Medicine, Ganga Medical Center and Hospital, Coimbatore, India; 2 Department of Orthopedics and Spine Surgery, Ganga Medical Center and Hospital, Coimbatore, India

**Keywords:** Saphenous nerve, Infrapatellar branch (IPBSN), Sartorial branch (SBSN), Inside-out, Outside-in, Paraesthesia, ACL reconstruction

## Abstract

**Background:**

Paraesthesia after hamstring graft harvest is a ubiquitous complication in the early post-operative period, and its correlation with vertical versus horizontal skin incision are well documented. The purpose of the study is to evaluate the incidence and extent/area of sensory loss of saphenous nerve branches occurring with the outside-in (OI) versus inside-out technique (IO) of semitendinosus graft harvest from the sartorius fascia and to determine a better method of graft harvest.

**Methods:**

Sixty patients who underwent isolated semitendinosus graft harvest during anterior cruciate ligament reconstruction (ACLR) between 2016 and 2017. Patients were randomised into two groups depending on the graft harvest technique: 30 in the OI group and 30 in the IO group. The area of sensory loss was mapped on the patients’ skin using tactile feedback from the patients at each follow-up (10 days, 1 month, 3 months, 6 months and 1 year). Then, the area of sensory changes for the infrapatellar branch (IPBSN) and sartorial branch (SBSN) of the saphenous nerve, incision length, graft harvest duration, and graft length were analysed statistically between the groups.

**Results:**

In groups 1 and 2, 18/30 (60%) and 19/30 (63%) of patients, respectively, developed sensory changes, with no significant difference between the groups (*p* = 0.79). Isolated SBSN and IPBSN paraesthesia occurred in 2/60 (3%) and 19/60 (32%), respectively. Combined SBSN and IPBSN paraesthesia was present in 16/60 (27%) of patients. There was no significant difference in the area of the sensory deficit between OI and IO groups on the 10th post-operative day or at 1-month, 3-month or 1-year follow-up (*p* = 0.723, *p* = 0.308, *p* = 0.478, *p* = 0.128, respectively). However, at 6-month follow-up, the area of paraesthesia was significantly higher in the IO group (*p* = 0.009). The length of incision and duration of graft harvest was higher in the OI group than in the IO group (*p* = 0.002 and *p* = 0.007, respectively), and the total length of the graft was greater in the IO group (*p* = 0.04).

**Conclusion:**

Incidence is equally distributed, area of iatrogenic saphenous nerve injury gradually decreases, and recovery is seen in the majority of the patients in both graft harvest techniques. IO graft harvesting technique is better in terms of graft harvest time and cosmetics and yields longer graft; however, area of paraesthesia, though not significant, was two-fold higher than the OI technique at 1-year follow-up.

**Clinical relevance:**

IO graft harvest technique would enable the surgeon to adopt quicker graft harvest, smaller surgical scar and lengthier graft than the OI technique.

**Level of evidence:**

Therapeutic randomised controlled prospective study, Level II.

## Introduction

Hamstring graft is a safe and effective alternative to patellar tendon autograft with comparable clinical outcomes. It has a lower rate of anterior knee pain in anterior cruciate ligament (ACL) reconstruction [1, 2]. The most prevalent post-operative symptom in patients undergoing ACL reconstruction is numbness in the leg compartment. After exiting the adductor canal, the saphenous nerve promptly divides into two terminal branches: the infrapatellar branch (IPBSN) and the sartorial branch (SBSN). The IPBSN and its inferior trunks supply the anteromedial aspect of the knee. At the same time, the SBSN takes a vertical course and travels down the medial knee behind the sartorius tendon in close association with the gracilis over a length of a few centimetres before becoming subcutaneous by piercing the fascia. Though hamstring graft is relatively safe to harvest, iatrogenic injury to the saphenous nerve branches (IPBSN and SBSN) may result in neuromas, reflex sympathetic dystrophy and anterior knee pain. These symptoms lead to patient dissatisfaction [3–5]. Studies have reported vulnerability of the isolated IPBSN injury in 37–86% of the cases [6] and combined IPBSN and SBSN injuries in 32% cases [7]. Further, the current literature recommends an oblique incision over the skin to reduce iatrogenic nerve injury to knee [8–10] as the nerve runs parallel to the incision. The posterior graft harvesting technique has lower sensory deficits [11]; however, technical difficulties and poor graft dimensions (shorter length and diameter of grafts) preclude this harvest.

Harvesting the semitendinosus graft has a lower risk of injury to branches of the saphenous nerve than harvesting both the hamstring grafts (semitendinosus and the gracilis) [12]. Transection of the nerve can occur during deep skin incision or when opening the pes anserine fascia, or during the release of accessory insertions and/or during blunt trauma to the nerve while passing a tendon stripper. Iatrogenic injury is due to the proximity of the saphenous nerve and its branches to the hamstring grafts or due to variable tendon morphology and variable nerve course.

The most prevalent technique of hamstring graft is the outside-in (OI) technique [13, 14] technique, where a small nick is made in the pes fascia, and the tendon is hooked out/pulled out with right-angled blunt artery forceps, without detaching pes anserine fascia. The other method is the inside-out (IO) technique [15, 16], where the tendon and pes fascia are detached along with the periosteum from the proximal tibia to harvest the graft. To study the iatrogenic nerve injury patterns, most authors have examined the skin incisions (horizontal versus vertical incision) [17, 18] over proximal tibia. However, to date, no studies have compared the sensory outcome of OI versus that of the IO technique of semitendinosus tendon graft harvest in terms of IBSN and SBSN nerve injury. Our primary aim is to compare and evaluate the incidence and extent/area of sensory loss of saphenous nerve branches in OI versus the IO technique of semitendinosus graft harvest. Our secondary aim is to determine a better method of graft harvest. We hypothesise that the IO technique is better than the OI technique with regard to iatrogenic nerve injury, time to harvest graft and length of skin incision.

## Material and method

**Fig. 1:**
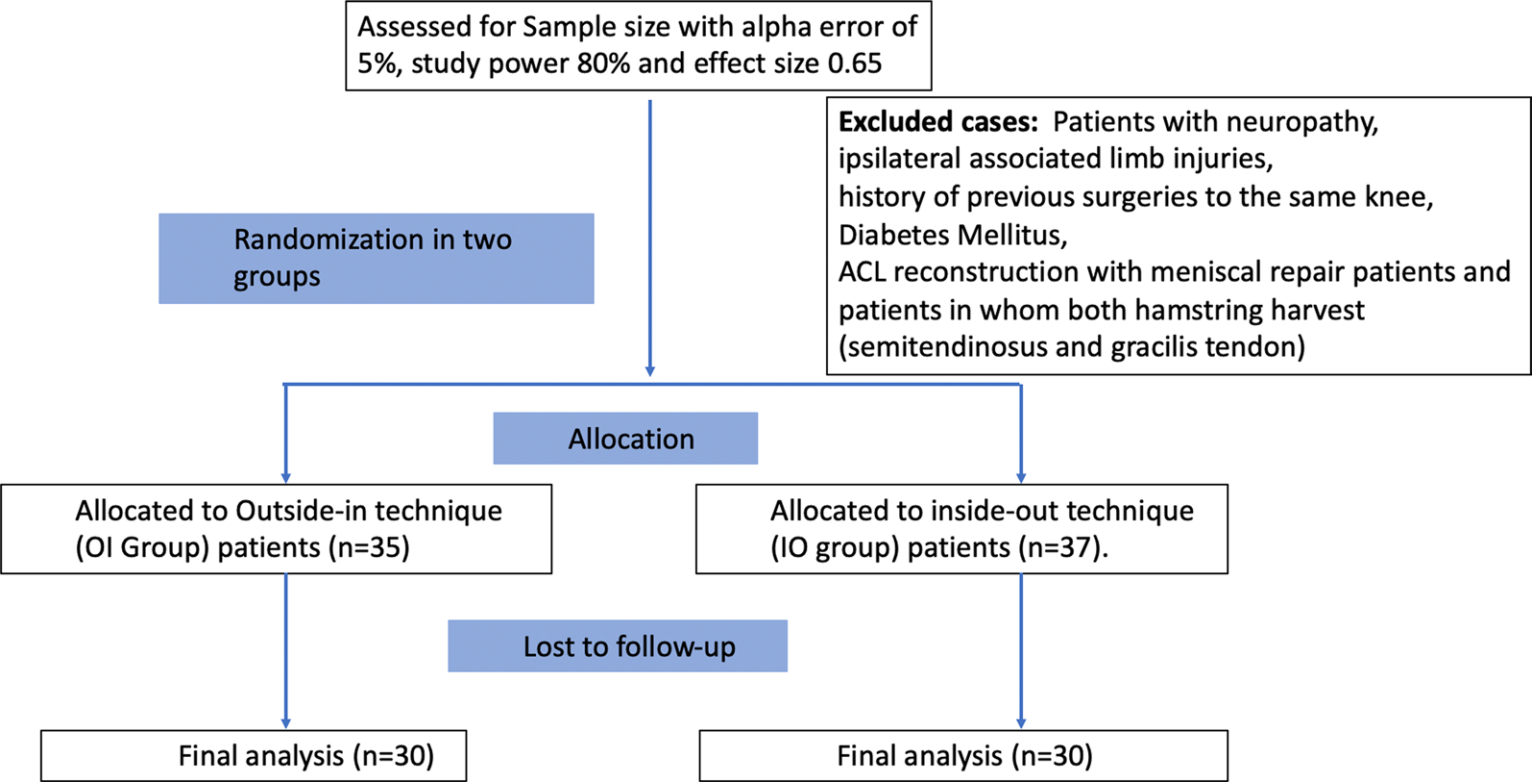
Flow diagram showing patient enrolment

All patients who underwent arthroscopic ACL reconstruction with semitendinosus graft between 2016 and 2017 were considered for this prospective randomised study. Using computer-generated randomisation, the patients were allocated into the OI group (*n* = 30) or the IO group (*n* = 30) on the basis of hamstring graft harvest technique. Ethical committee and institutional review board (IRB) approval was obtained for this study (IRB number 2016/02/04). Patients aged between 18 and 50 years who underwent arthroscopic ACL reconstruction with a single graft (semitendinosus) were included (Fig. 1). Patients with neuropathy, associated limb injuries, history of previous surgeries to the same knee, diabetes mellitus or ACL reconstruction with meniscal repair, and those in whom both the semitendinosus and gracilis tendon was harvested, were excluded from the study. Informed consent was obtained from all participants. The graft was harvested by a single senior surgeon (S.R.S.) in all patients.

### Surgical technique and rehabilitation protocol

All patients were operated on under spinal anaesthesia, and post-operatively ultrasound-guided adductor canal block was given for post-operative pain relief. Arthroscopic ACL reconstruction was performed with a single graft (semitendinosus) in all cases. In both techniques of hamstring graft harvest, an oblique skin incision was made, 2 cm below and medial to the tibial tubercle.

#### Outside-in (OI) graft harvest

**Fig. 2:**
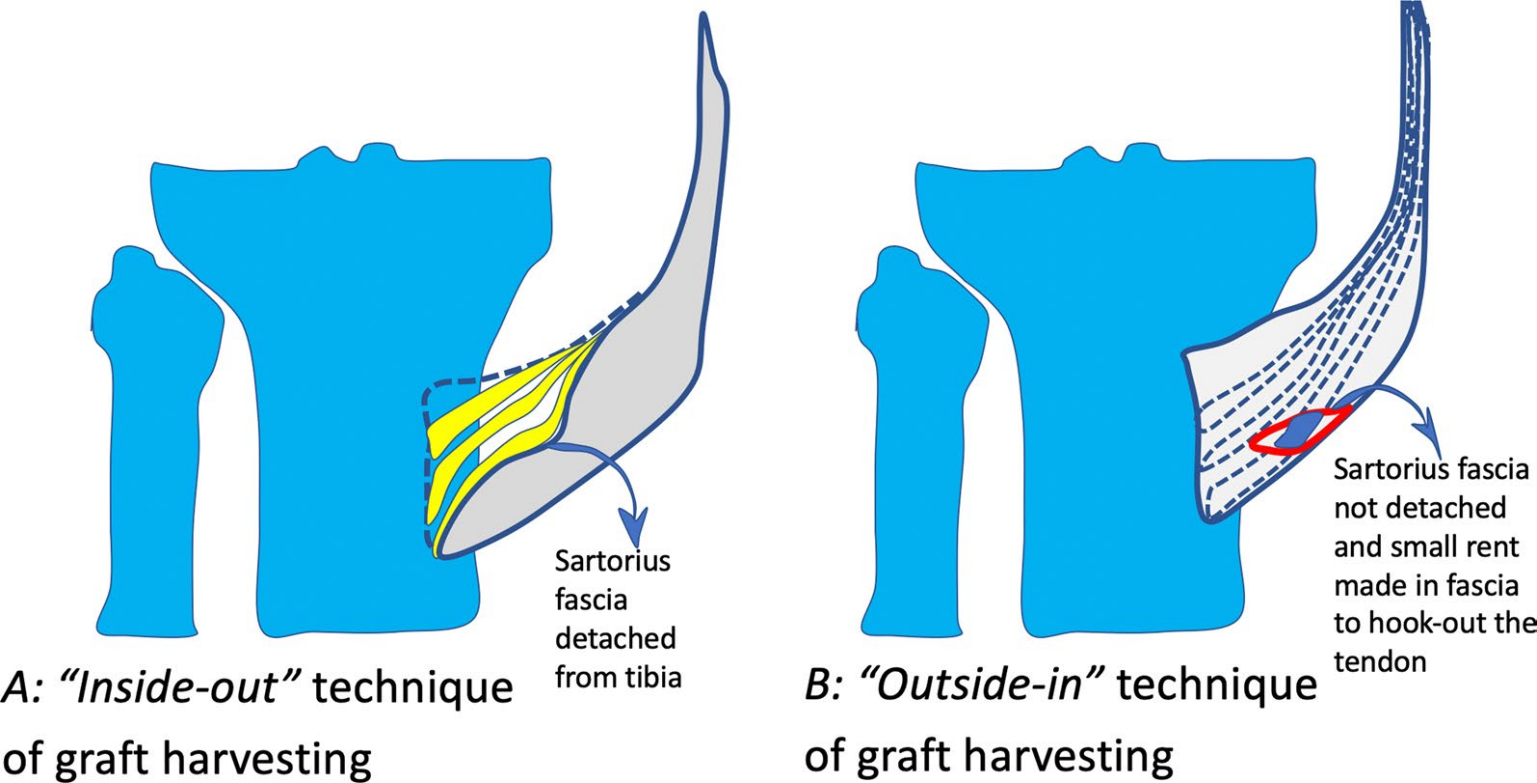
Pictorial representation of the two techniques: **A** IO technique of graft harvesting, where sartorius fascia is detached from the tibial insertion, and semitendinosus (ST) is separated from the fascia and harvested; **B** OI technique where sartorius fascia was not detached from the tibia and a small nick was made in the fascia to hook out the ST tendon and harvest it

Pes anserine fascia was not detached from the tibia in this technique. The fascia was opened via a horizontal nick on the outer aspect of the fascia (Fig. 2A), then the gracilis was retracted and semitendinosus was identified. The semitendinosus was then hooked out of the fascia with 90° artery forceps, and the vincula were released using Metzenbaum scissors one after another. The tibial expansions were cut, and the tendon was detached from the bone. The tendon was harvested using a closed tendon stripper. The graft was then prepared, and the total length of the graft was measured and documented.

#### Inside-out (IO) graft harvest

In the IO technique, pes anserine fascia detached along with proximal tibial periosteum by a meticulous technique of scraping the tibial periosteum along with fascia and the graft. The semitendinosus graft is visualised from the inner aspect of the fascia, and it is separated from the fascia. Then the graft is pulled out of the wound, and the vincula are released using Metzenbaum scissors. The tendon was harvested using a closed tendon stripper. Then, the graft was prepared, and the total length of the graft was measured and documented. After ACL reconstruction, the pes anserine fascia was sutured to the tibia. Patients were informed on the institutional rehabilitation protocol in both groups. Patients were mobilised with crutches and weight-bearing as tolerated from the first post-operative day, and active-assisted knee movements were started on the second day. Full weight-bearing walking with crutches was allowed from the second week and progressed to walking without crutches from the third post-operative week. Active knee movements started in the second week, and full knee range of movements was achieved by 4 weeks. Strengthening exercises and half-squat exercises began in the 12th week. Sports, running and squatting on the floor were allowed only from the sixth post-operative month.

**Fig. 3:**
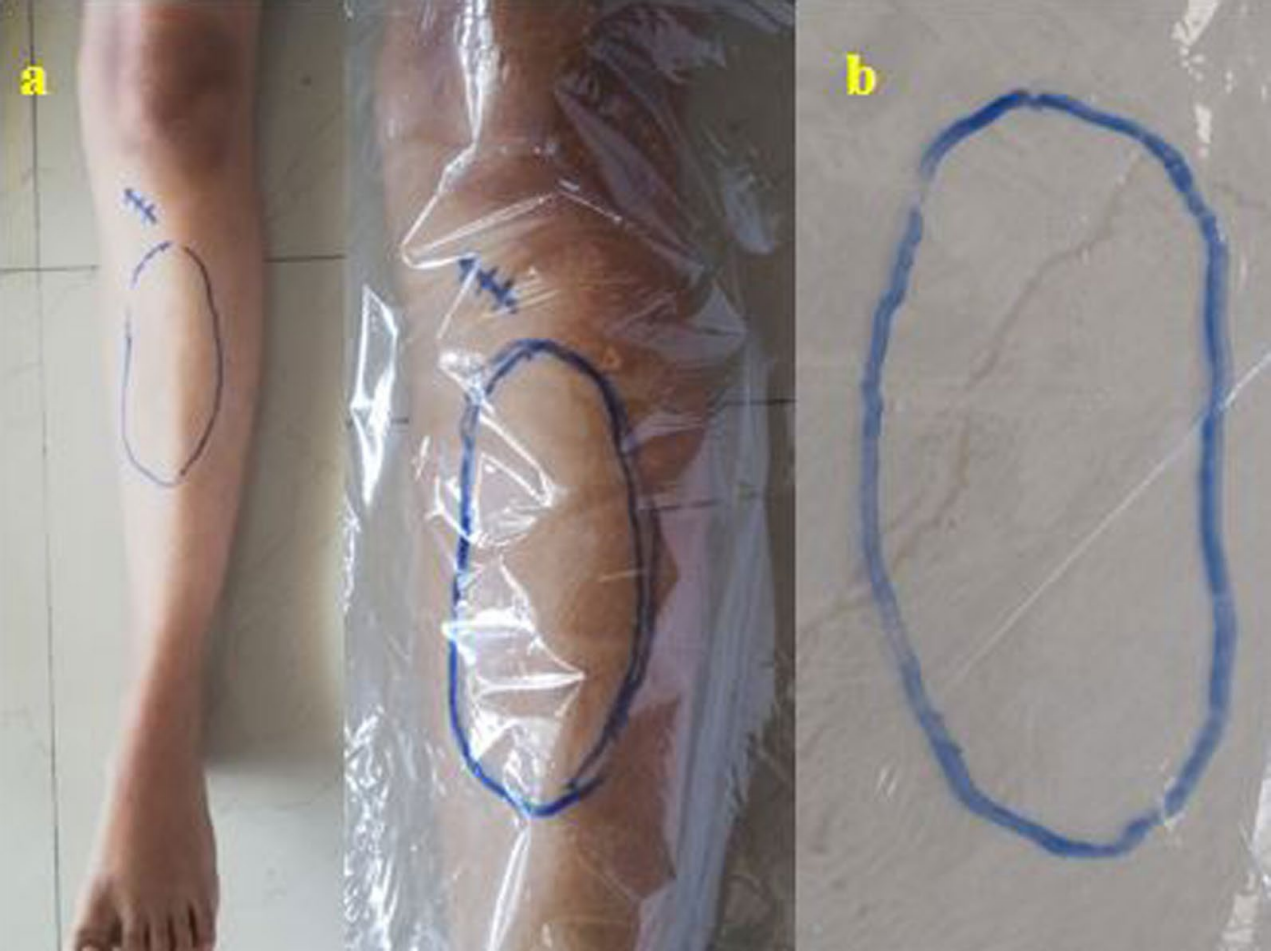
**a** Localisation and measurement of the area of paraesthesia over skin with tracing on a transparent plastic sheet, **b** Tracing sheet is photographed, and measurement is done with the help of Image J software

All the patients were clinically evaluated on the 10th post-operative day, then at 1, 3, 6 and 12 months of follow-up. The incision length was measured in centimetres, and the time taken to harvest the graft was recorded and documented. The subjective area of sensory loss was assessed for the involvement of SBSN, IPBSN and combined anatomical zones of these nerves. A blunt pin was used for pinprick examination starting from the superior pole of the patella then moved distally in all directions. The patient was asked to point out the change in sensation from altered to normal skin sensation, marked with a skin marking pen (Fig. 3). The adjacent area was identified within 0.5 cm of the previous boundary, and the process was continued until the skin over the entire leg length was mapped. All the demarcated points were joined with small straight lines and completed skin mapping. Then, a transparent polythene sheet of adequate size was placed over the mapped area on the skin, and a tracing was done (Fig. 3). The transparent sheet was laid over a calibrated grid-lined graph paper, and digital photographs of the paraesthesia area were recorded (Fig. 3). The extent of hypoaesthesia (cm^2^) was measured and analysed with Image J software of the digital photographs. The paraesthesia area was mainly evaluated for cutaneous nerve distribution of the saphenous nerve and its terminal branches in the leg compartment.

### Statistical analysis

For statistical analysis, we used SPSS software. The sample size was calculated by the G*power program, using a priori statistical power analysis as performed with an area of sensory loss at 1-month post-operative duration as a primary outcome measure, with an alpha error of 5%, study power 80% and effect size 0.65 (moderate to significant difference). Effect size is calculated by taking the difference between the two groups and dividing it by the standard deviation of one of the groups. The chi-squared test was used to compare the categorical variables between the two groups, and a paired *t*-test was used for continuous variables. A *p*-value of < 0.05 was considered statistically significant. The sample size calculation was done with power analysis for the primary variable for the incidence of nerve damage, area of paraesthesia, time taken to harvest graft, and length of skin incision. It was 0.04, 0.06, 0.9 and 0.9, for these primary variables. This confirms that the sample size is adequate for comparing time to graft harvest and skin incision length.

## Results

Sixty patients (53 male and 7 female) were enrolled as per inclusion and exclusion criteria with a minimum follow-up of 12 months. The right knee was involved in 42 patients, and the left knee in 18 patients. In this study, 37/60 (62%) of the patients developed sensory changes due to iatrogenic injury to branches of the saphenous nerve. Statistically, there was no significant (*p* = 0.79) difference between the two groups (Table 1). Isolated SBSN (3%), IPBSN (30%) and combined SBSN and IPBSN (27%) paraesthesia was seen among the study population, and there was no difference in nerve injury pattern between the OI group and the IO group (Table 2). From the clinical mapping, the area of sensory change was not significantly different between the groups on the 10th post-operative day or at 1 month, 3 months and 12 months of follow-up (*p* = 0.723, *p* = 0.308, *p* = 0.478, *p* = 0.128, respectively) (Table 3). However, the area of hypoaesthesia was significantly greater in the IO group than the OI group at 6 months follow-up (*p* = 0.009) (Table 3). There was a considerable improvement in hypoaesthesia from the 10th post-operative day to 6 months in both the OI and the IO group (*p* = 0.038, and *p* = 0.001, respectively). At 1 year follow-up, three patients in the IO group and four in the OI group had persistent paraesthesia, with complete recovery in the remaining patients. Further, the length of incision was greater in the OI group, and the duration of graft harvest was longer in the OI group than the IO group (*p* = 0.002 and *p* = 0.007, respectively), and the mean total length of the graft was longer in the IO group (*p* = 0.04) (Table 4). None of our patients has any neuralgia, graft site infection, chronic regional pain syndrome (CRPS), hyperalgesia, autonomic changes, trophic changes, oedema or functional loss.

**Table 1:** Number of patients with paraesthesia at different time periods during follow-up in two groups

Number of patients with paraesthesia	Outside-in technique OI group (*n* = 30)	Inside-out technique IO group 2 (*n* = 30)	*p*-Value
At 10th day	18	19	0.79 (n.s.)
At 1 month	18	19	0.79 (n.s.)
At 6 months	6	11	0.152 (n.s.)
At 12 months	4	3	0.161 (n.s.)

*n.s.* not significant

**Table 2:** Type of nerve injury in different groups

Type of injury	Outside-in technique (*n* = 18/30)	Inside-out technique (*n* = 19/30)	Total (*n* = 37/60)
IPSN	10	9	19 (31.7%)
SBSN	1	1	2 (3.3%)
Combined IPSN + SBSN	7	9	16 (26.7%)

**Table 3:** Area of paraesthesia (mean ± standard deviation (SD), cm^2^) at different time periods during follow-up in two groups

Time of follow-up	Outside-in technique (*n* = 30)	Inside-out technique (*n* = 30)	*p*-Value
At 10th day	148.94 ± 109.67	136.27 ± 103.8	0.723 (n.s.)
At 1 month	141.1 ± 107.3	123.7 ± 100.03	0.308 (n.s.)
At 3 months	143.7 ± 114.12	116.1 ± 110.8	0.478 (n.s.)
At 6 months	34.55 ± 62.43	103.92 ± 90.11	0.009
At 12 months	39.1 ± 16.4	89.3 ± 53.4	0.128 (n.s.)

*n.s.* not significant

**Table 4:** Patient characteristics in the study

	OI group	IO group	*p*-Value
Age (years) mean ± SD	32.9 ± 11.5	33.59 ± 9.75	
Side (left/right)	10/18	8/22	
Male/female	27/3	27/3	
Length of skin incision (cm) Mean ± SD	4.83 ± 0.69	3.93 ± 0.65	0.002
Time taken to harvest graft (min) Mean ± SD	3.6 ± .994	2.8 ± .887	0.007
Total length of the graft, mean ± SD	27.3 ± 1.49	29.7 ± 2.1	0.04

## Discussion

The most important finding of this study is that the incidence of paraesthesia was equally distributed between the groups. Although the area of paraesthesia was twice as large in the IO group than in the OI group at 1-year follow-up, the difference was not statistically significant. However, the IO technique enabled quicker and lengthier semitendinosus graft harvest with a lesser length of skin incision.

Patients’ expectations for ACL reconstruction have increased over the years, and knee pain after ACLR has been attributed to injury of the IPBSN branch [19]. Tennent et al. demonstrated the anatomy of saphenous nerves and their branches in the cadaver specimen, and correlated surgical intervention with possible iatrogenic injuries to these nerves. In our series, most of the patients had numbness and paraesthesia. We found that the incidence of paraesthesia (in OI and IO groups) was due to iatrogenic nerve injury in 62% of cases. This observation was similar to that of Mochizuki et al. [20], who observed sensory disturbances in 58% of their patients in whom medial hamstring tendons were harvested with a vertical incision. However, they did not compare the sensory distribution of the two harvest techniques. Other authors have reported variable rates of post-operative hypoaesthesia, ranging from 30% to 77% [2, 6, 9, 13, 21–23].

Aglietti et al. [24] reported an average area of sensory loss of 25 cm^2^, and Sipahioglu et al. [25] found that the area of hypoaesthesia was significantly higher in the group undergoing vertical skin incision (42.4 ± 22.3 cm^2^) compared with oblique skin incision (9.3 ± 15.3 cm^2^). Unlike their study, we compared the area of paraesthesia for graft harvest technique (OI versus IO technique) rather than skin incision. In our study, the average area of sensory loss was significantly larger in the IO group than in the OI group at 6-month follow-up (103.92 ± 90.11 cm^2^ in the IO group versus 34.55 ± 62.43 cm^2^ in the OI group; *p* = 0.009). There was a drastic reduction in the area of sensory loss in the OI group, and a gradual reduction in the IO group, which explains the significant difference at month 6. This is due to the technical aspect of harvesting in the OI group, where the sartorius fascia is not being detached; it is possible that recovery is faster with OI graft harvest technique. However, at 1-year follow-up, though the area of paraesthesia in the IO group was twice as large as in the OI group, there was no statistically significant difference between the groups. The clinical implication is a gradual reduction in the sensory loss in both groups that could continue even after 1 year of follow-up. This is supported by Zhu et al. [26], who reported that the area of hypoaesthesia gradually decreases with time and even recovers completely. Various studies [18, 26–28] have reported the area of sensory loss for different methods of skin incision, but this is the only study to have assessed sensory changes by two different techniques of graft harvest [by detaching sartorial fascia (IO group)/without detachment (OI group)]. In this study, an oblique skin incision was used in both groups since this incision is associated with less iatrogenic nerve injury/fewer complications, as reported in anatomical studies [27–29]. Ruffilli et al. [10] in their systematic review concluded that adaptation to an oblique incision over skin would cause less neurological impairment. However, no studies considered the method of hamstring graft harvest (IO vs OI techniques) to assess neurological impairment. In this study, an oblique incision was made 2 cm below and medial to the apex of the tibial tuberosity. Traditionally, hamstring graft has been harvested by two surgical techniques, with or without detaching pes anserine/sartorius fascia from the proximal tibia. Further, from cadaveric study, it is evident that IPBSN and SBSN nerves ramify in a close relationship with sartorius fascia. Hence, this comparison (IO versus OI technique) would be more insightful in assessing sensory changes after graft harvest.

The pattern of iatrogenic injury in our study was similar to that observed by Sanders et al. [7], who mapped nerve involvement for combined nerves (IPBSN with SBSN) as well as isolated IPBSN and SBSN. However, unlike our study, Sanders et al. reported isolated SBSN nerve involvement in 23% and isolated IPBSN in 19% of cases. This difference may be due to the fact that we harvested the graft with more than 120° knee flexion, which reduces the iatrogenic injury to the nerve, where the SBSN courses posterior to the skin incision and the pes anserine/sartorial fascia. Dunaway et al. have substantiated that the nerve courses more posteriorly with increased knee flexion [30].

The residual paraesthesia in our study was 19% at 1-year follow-up; on the contrary, Spicer et al. [31] reported permanent sensory loss in nearly 50% of cases after 2 years. However, unlike our study, all their patients underwent vertical incisions with both the grafts (semitendinosus and gracilis graft) being harvested, which increases the chance of nerve injury. Thus, single graft harvest (semitendinosus alone) and oblique skin incision would be a wise option to reduce the morbidity due to graft harvest.

Adequate graft length is necessary for single graft (semitendinosus alone) reconstruction without compromising on fixation [32]. IO graft harvesting has advantages as the semitendinosus insertion part of the graft is visualised from the inner aspect of the fascia, unlike the OI technique, where the tendon is hooked out before detachment. Hence, the IO technique has the added advantage of harvesting the graft’s additional length (about 1–1.5 cm of proximal tibial periosteum can be detached along with graft) with the meticulous technique of scraping tibial periosteum along with the graft during detachment. This is evident from the IO technique yielding better graft length after the harvest in this study. Primarily, this is very useful in single tendon graft harvest, as it allows better graft dimensions (graft length). This observation was similar to that of Pagnani et al. [13], who measured the insertion point of the conjoined structure on the anteromedial tibial surface and reported a mean location of 1.9 cm distal to and 2.25 cm medial to the apex of the tibial tuberosity. Hence, harvesting the graft by the IO technique by detaching the graft along with the periosteum is advantageous in single graft ACL reconstructions, and in the OI technique, this would not be possible. Thus, the IO technique has all the prerequisites for a good graft harvest.

Khanna et al. [33] suggested that posterior hamstring harvest allows for a more rapid harvest. In their series of 214 patients, no one expressed any cosmetic concerns about the incision. However, they did not assess the time to harvest in their study. In this study, we observed a significant difference in the time to harvest in the IO group compared with the OI group. Opening the sartorius fascia made it easier to identify the semitendinosus graft and achieve a quicker harvest in the IO group, whereas in the OI group, tactile feedback was necessary before making a nick on the outer layer of sartorius fascia, and semitendinosus had to be hooked out, detached and harvested. Hence, the OI technique led to a longer time to harvest than in the IO group, and in obese patients, it was much more prolonged. Further, the length of incision needed was significantly smaller and cosmetics were better in the IO group than the OI group. No other study has reported the time to harvest and cosmetic concerns due to graft harvesting after skin incision.

Limitations of our study include that the observations were made by a single surgeon and the follow-up period was short. Moreover, limited cohort and subgroup analysis could not be carried out as the sample size was insufficient to draw robust statistical conclusions. Additional assessments, that is, an electrophysiological study, would have been helpful. Fortunately, the clinical ramifications of electrophysiological events are usually low, and most nerve injuries do not result in much disability/morbidity. However, neurovascular injuries can have medico-legal implications; thus, it is essential to understand that nerve injury may be an inherent problem associated with mini-incision and distal-to-proximal harvest. Patients should be informed about this event.

## Conclusion

Incidence is equally distributed, area of iatrogenic saphenous nerve injury gradually decreases, and recovery is seen in the majority of patients in both techniques of graft harvest. IO graft harvesting technique was better in terms of graft harvest time and cosmetics and yielded longer graft; however, area of paraesthesia, though not significant, was two-fold higher than OI technique at 1-year follow-up.

## Data Availability

Available.
